# Structural analysis of hierarchically organized zeolites

**DOI:** 10.1038/ncomms9633

**Published:** 2015-10-20

**Authors:** Sharon Mitchell, Ana B. Pinar, Jeffrey Kenvin, Paolo Crivelli, Jörg Kärger, Javier Pérez-Ramírez

**Affiliations:** 1 ETH Zurich, Department of Chemistry and Applied Biosciences, Institute for Chemical and Bioengineering, Vladimir-Prelog-Weg 1, 8093 Zurich, Switzerland; 2 ETH Zurich, Department of Materials, Laboratory of Crystallography, Vladimir-Prelog-Weg 5, 8093 Zurich, Switzerland; 3 Micromeritics Instruments Corporation, Communications Drive 4356, Norcross, Georgia 30093-2901, USA; 4 ETH Zurich, Department of Physics, Institute for Particle Physics, Otto-Stern-Weg 5, 8093 Zurich, Switzerland; 5 University of Leipzig, Linnestrasse 5, 04103 Leipzig, Germany

## Abstract

Advances in materials synthesis bring about many opportunities for technological applications, but are often accompanied by unprecedented complexity. This is clearly illustrated by the case of hierarchically organized zeolite catalysts, a class of crystalline microporous solids that has been revolutionized by the engineering of multilevel pore architectures, which combine unique chemical functionality with efficient molecular transport. Three key attributes, the crystal, the pore and the active site structure, can be expected to dominate the design process. This review examines the adequacy of the palette of techniques applied to characterize these distinguishing features and their catalytic impact.

## Abstract (web summary)

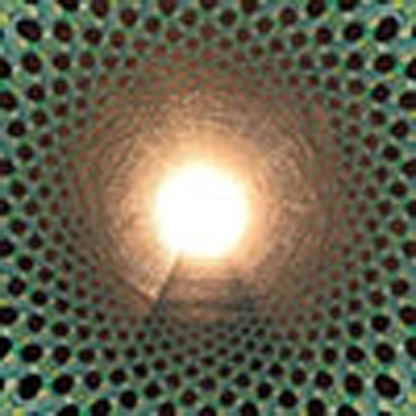
Hierarchically organized zeolites are materials retaining the crystalline order and associated functionality of bulk zeolites while also integrating a multilevel pore network. Here, the authors review the raft of techniques applied to characterize their crystal, pore and active site structures.

Inspired by nature's elegant examples and proclaimed as the next frontier in advanced materials design, ‘hierarchical organization' has become a truly interdisciplinary phenomenon in the development of new technologies^1^,^2^,^3^,^4^,^5^,^6^. While the various uses of the quoted term are often considered as closely related, in materials science, it denotes the engineering of one or more phases to exploit structural order on different length scales^1^,^2^. The latter is pursued to enhance complementary characteristics (mechanical, thermal, electrical, optical, mass transfer and so on) and has the potential to bestow a performance unimaginable of the bulk constituents.

A field in which the development of hierarchically organized materials has experienced exceptional progress, having proven industrial relevance and reached advanced stages of the design, is that of zeolite catalysis^5^,^6^,^7^,^8^,^9^,^10^,^11^,^12^,^13^,^14^,^15^,^16^. In this context, a hierarchically organized zeolite (HOZ) is defined as a material that retains the crystalline order and associated functionality of a bulk (purely microporous) zeolite, but that also integrates a multilevel pore network. Analogous to improving the traffic circulation by introducing wide freeways along directions of major transit in cities, this centres on the introduction of an interconnected network of auxiliary meso- and/or macropores to enhance molecular transport in reactions in which diffusion is constrained within the micropores, which are typically of 0.3–1 nm in diameter. In this way, reactants and products can readily enter and leave the microporous domains, thereby maximizing the utilization of the active sites throughout the entire catalyst volume. The additional porosity levels can be configured either within (intracrystalline) or between (intercrystalline) the zeolite crystals, effectively shortening the diffusion path inside the micropores in both cases. This definition is further extended by the fact that additional phases may also be incorporated to achieve the desired porosity characteristics, such as in the case of inorganic or organic pillars, forming the basis of an endless spectrum of structural variants combining differing types, degrees and distributions of secondary porosity (Fig. 1).

The logical question follows: how can this information aid the design of a superior zeolite catalyst? Catalytic evaluation of HOZs has demonstrated numerous opportunities for enhanced performance in both traditional and emerging applications^5^,^6^,^7^. However, despite this extensive repertoire, for every competitive advantage there typically lies a threat. For example, as expected due to the increased external or mesopore surface area, HOZs are generally more active than their bulk predecessors in diffusion-constrained reactions, such as those involving the transformation of larger substrates or those undertaken in the liquid phase (Fig. 2a,b). The achievable enhancement strongly depends on the extent of mass transfer limitations and can exceed an order of magnitude^7^,^15^,^16^,^17^,^18^. Yet, this assumes that the active sites remain accessible and of similar quality. Depending on the acidity demands of the reaction, it has been shown that reductions in the strength and/or concentration of acid sites, which often accompany losses of the crystalline order, can impair or even reverse the catalytic benefits^16^,^17^,^18^. A similar case can be argued with respect to selectivity, where both beneficial and detrimental impacts have been evidenced over HOZs. Moreover, in chemical transformations involving multiple steps, different impacts may be observed on the selectivity to primary and secondary products^17^. Improvements are typically ascribed to the more efficient transport of the desired product out of the zeolite crystal^9^,^16^, while deteriorations are related to a loss of the shape-selective properties either because the diffusion path within the micropores is too short or due to the increased number of unselective active sites present at the external or mesopore surface (Fig. 2c)^7^,^17^,^19^. Finally, in terms of stability, the increased mesopore surface area in HOZs is known to significantly retard the rate of reversible deactivation in reactions suffering from prevalent coke deposition (Fig. 2d)^20^,^21^,^22^. On the other hand, reduced catalyst lifetimes have also been evidenced, which were ascribed to a compromised acidity and the poor quality of the auxiliary pore network^22^.

The above analysis points overwhelmingly towards the need for the application oriented design of HOZ catalysts, Three key attributes can be expected to dominate this process: the crystal, the pore and the active site structure. To date, the development and refinement of numerous synthetic protocols that enable the preparation of HOZs of any framework type or composition has been documented^5^,^6^,^7^,^8^,^9^,^10^,^11^,^12^,^13^. However, despite going hand in glove with progress in their synthesis, no review has previously examined the structural analysis of HOZs. The latter presents a far greater challenge than that of their bulk predecessors and is essential to rationalize their catalytic performance. For this purpose, the array of state-of-the-art techniques that have been applied to assess each of these parameters (Table 1) are critically examined, outlining the achievements and limitations and providing directions to push the boundaries of current approaches, respectively.

## Synthesis mechanism

While this review does not comprehensively overview recent advances in the synthetic methodologies of HOZs, to understand their properties it is helpful to consider some important aspects regarding their synthesis. Here we focus on the fundamental question: what is known about the mechanism of hierarchical structuring of the porosity of a zeolite? Interrupting or suppressing the crystal growth in a given dimension and controlling the assembly during the synthesis, or dissolving the wall of a bulk zeolite by post-synthetic modification, have consequences that need to be understood. This can be considered at different length scales, both in terms of the resulting location and topology of mesopores and of the impact on the crystal and associated active site structure.

**Figure 3:**
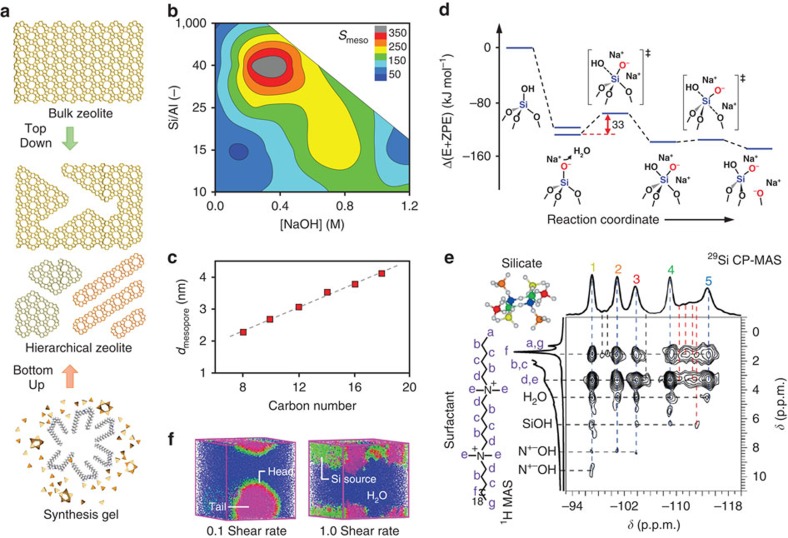
Mechanism of HOZ synthesis. (**a**) Approaches to synthesize HOZs involve either interrupting their crystallization and growth in the synthesis gel (bottom-up) or the post-synthetic removal or rearrangement of framework atoms in a bulk zeolite (top-down). A multilevel mechanistic understanding is vital to effectively control the crystal, active site and pore structure. For top-down methods, (**b**) a contour plot of the mesopore surface area of demetallated ZSM-5 zeolites versus the base concentration was applied and the starting Si/Al ratio and **c**, the linear dependence between the average mesopore diameter and the surfactant length in the post-synthetic mesostructuring of zeolite Y, typify the mesoscopic observations attained experimentally. (**d**) As illustrated by the zero-point energy-corrected energy profiles (E+ZPE), theoretical efforts to improve understanding at the atomic level have so far focused on predicting the likely first steps of demetallation pathways. Regarding direct synthesis methods (**e**), unparalleled atomic-level insights have been attained by solid state NMR. Here, 2D ^29^Si {^1^H} heteronuclear shift correlation (HECTOR) experiments reveal strong intensity correlations between the head groups of diquarternary ammonium surfactants and the resulting ZSM-5 nanosheets and the nanolayered silicate intermediates. (**f**) Dissipative particle dynamics simulations of the impact of the shear rate on the self-assembly of an amphiphilic surfactant and silica source in aqueous solution represent one of the first computational contributions to the mesoscopic understanding. (**b**–**f**) Adapted with permission from ref. 23 (**b**, © 2011 The Royal Society of Chemistry), 15 (**c**, © 2014 John Wiley and Sons Inc.), 33 (**d**, © 2014 American Chemical Society), 39 (**e**, © 2014 John Wiley and Sons Inc.) and 43 (**f**, © 2015 Elsevier).

The mechanistic diversity can be appreciated by considering that HOZs can be synthesized by essentially opposite, bottom-up versus top-down, approaches (Fig. 3a). To date, the synthesis has predominantly been optimized through the experimental monitoring of key variables (Fig. 3b,c)^15^,^23^. Yet, while providing a mesoscopic framework through which to tackle the design, as with any trial and error approach, the finesse with which this can be accomplished depends on the comprehensiveness of the initial screening, and deviations from the established trends are highly probable. A clear example is found in the case of post-synthetic demetallation. In particular, the method of desilication in alkali media, which has been extensively studied due to its combined industrial readiness and versatility^14^,^23^, has experienced several paradigm shifts regarding the scope. For example, it took over 5 years to vanquish the initial dogma that the applicability was limited to a narrow compositional window of zeolites with Si/Al ratios of 25–50 (refs 24, 25). Similarly, the potentially key role of defects, which to date has remained rather obscure^26^, recently resurfaced when it was demonstrated that a defect free MFI-type zeolite was stable in alkaline media for up to 1 week^27^. The critical impact of the manufacturing conditions on the mesopore location and associated ability to enhance the transport attributes has also only lately been established^28^.

Progress in the understanding of how mesopore formation propagates through the crystal during demetallation, and the structure and functionality of the newly created mesopore wall, remains limited. Except in the case of processes targeting the hydrolytic extraction of more labile elements as boron or germanium located in crystallographically defined sites^29^,^30^,^31^, as has been elegantly exploited by Roth *et al.*^29^ through the ADOR (assembly–disassembly–organization–reassembly) approach, it is unclear at the molecular level which and why certain lattice positions and framework types are more susceptible to demetallation than others, what defects are created, as well as whether extraframework species form or may be reorganized by the treatment. Experimentally, the main obstacles are the detection limit and spatial sensitivity of existing characterization tools as nuclear magnetic resonance (NMR), X-ray diffraction (XRD) and extended X-ray absorption fine structure. On the other hand, the theoretical understanding remains in early stages. Only a handful of density functional theory studies have taken pioneering steps to calculate the potential hydrolysis pathways of desilication and dealumination and the structure of the resulting extraframework species, sometimes leading to surprising findings (Fig. 3d)^32^,^33^,^34^,^35^,^36^. For example, in the first study of the alkaline treatment of MFI-type zeolites, Zhai *et al.*^33^ reported that dealumination was more energetically favourable than desilication, but that leached aluminium species rapidly readsorbed on the zeolite. This would contradict the view based on experimental observations that aluminium exerts a stabilizing effect on the framework^24^,^25^. Nonetheless, the real processes are certainly more complex than those simulated, and a more exhaustive study of the elementary steps is required to gain confidence in the findings. In this respect, the large unit cells of zeolites and the presence of several water molecules and ions pose major challenges, which is why most works have only considered the removal of one or two atoms in the gas phase^32^. Taking into account collective effects arising due to the dynamic nature of the system and understanding the mesoscale propagation of the mesopores will constitute key milestones in future work.

Comparatively, an equally abundant list of questions can be elaborated for the direct synthesis of HOZs, which invariably is achieved through the application of variable quantities of specific templating species of different sizes and functionality^37^,^38^,^39^,^40^,^41^,^42^. One of the first challenges is to predict the complex behaviour of templating species within the synthesis gel. Towards this goal, most efforts have focused on characterizing the stepwise transformations occurring in relation to the diverse morphologies of the resulting zeolites. The atomic interaction of the template with framework metals has also been confirmed by solid state NMR (Fig. 3e)^39^,^40^,^41^,^42^. In an alternative approach, Ren *et al.*^43^ recently demonstrated a mesoscopic simulation approach that could open a window for investigating the formulation of hierarchical zeolites prepared by surfactant-driven routes (Fig. 3f). However, researchers still struggle to predict exactly how and to what extent a template will suppress the crystal growth and control the assembly and intergrowth behaviour of the resulting nanocrystals. Another bottom-up method that has seen growing interest is that of repetitive branching, in which HOZs in the form of intergrown (self-pillared) nanosheets are attained by introducing morphology modifying additives to induce intergrowth structures^44^,^45^,^46^,^47^. Advantageously for their technical potential, Inayat *et al.*^47^ recently demonstrated the possibility to attain zeolites with similar morphologies using readily available inorganic salts. The major importance of controlling zeolite nucleation, which has also enabled the template free preparation of three-dimensional (3D) nanosized EMT- and FAU-type zeolites^48^,^49^, indicate that an improved understanding of the synthesis and properties of HOZs will undoubtedly benefit the manufacturing efficiency and performance of these materials.

## Crystal structure

The defining feature of a zeolite is a crystalline microporous framework, which originates its unique catalytic functionality. Indeed, the microenvironments defined by the periodic extension of interconnected channels and cavities impart the shape selectivity that is now a strong hallmark of zeolite catalysis. Yet, in endeavouring to reduce the diffusion path length within the micropores, the design of HOZs can stretch this definition to its limit. Apart from diminishing the size of the crystal domains, the introduction of auxiliary pore networks can result in increased defect concentrations or amorphization of the framework. Thus, the crystal structure is one of the first characteristics assessed in the preparation of HOZ catalysts.

**Figure 4:**
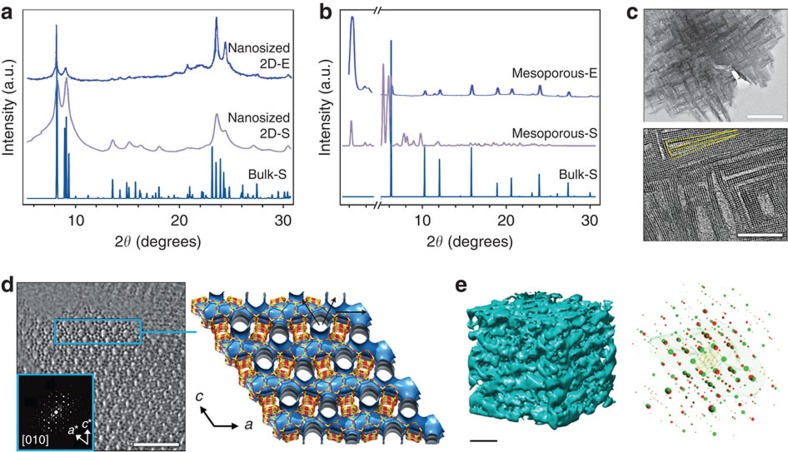
Crystalline structure of HOZs. Simulated (-S) and experimental (-E) XRD patterns of (**a**) a 2D nanosized ZSM-5 zeolite and (**b**) a USY zeolite with hexagonally ordered mesopores compared with the simulated patterns of their respective bulk forms illustrate the changes that can be expected upon reducing the crystal size in one or more dimensions. In the first case, only the 0*kl* reflections are distinguished, while in the latter case, low-angle reflections appear, and depending on the relative integration of the micro- and mesopore domains, high-angle reflections arising from the crystalline framework may be significantly altered, making it hard to identify with respect to the bulk phase. (**c**–**e**) Electron-based techniques are widely applied to gain complementary information on the crystal structure. (**c**) TEM images confirm the morphology and crystalline order of a 2D nanosized MFI-type zeolite, revealing the 90° rotational boundaries between the nanosheets (scale bars, 200 nm, top of panel; 20 nm, bottom of panel). (**d**) Electron crystallography enables the structure determination of a complex intergrown ITQ-39 zeolite (scale bar, 5 nm in TEM image). (**e**) The reciprocal lattice (right) reconstructed from rotation electron diffraction data confirms the preserved crystalline order of a hierarchically organized FAU zeolite. A 3D reconstruction of the pore topology from TEM tomography data (left) evidences the abundant presence of interconnected mesopores (scale bar 10 nm). (**a**–**e**) Adapted with permission from ref. 50 (**a**, © 2015 Macmillan Publishers Ltd), 51 (**b**, © 2012 The Royal Society of Chemistry), 42 (**c**, © 2014 Macmillan Publishers Ltd), 55 (**d**, © 2012 Macmillan Publishers Ltd) and 56 (**e**, © 2014 John Wiley and Sons Inc.).

While the initial structure elucidation relies upon X-ray powder diffraction, the reduced number of lattice planes to produce constructive interference in one or more directions means that the diffraction patterns of HOZs can become unrecognizable from those of bulk zeolites of equivalent framework type. This is clearly illustrated by comparing the simulated and experimental diffraction patterns of different types of HOZs (Fig. 4a,b). In the first example, the thickness of the crystal is reduced to that of a single unit cell in one direction, representative of the case of a two-dimensional nanosized zeolite^50^, and consequently no reflections with a component perpendicular to the layer are visible. On the other hand, if the size of the crystals is reduced to only a few unit cells in any direction, as in the case of a 3D nanosized zeolite, considerable broadening of all reflections would be expected. Another extreme case is that of ordered mesoporous zeolites in which the mesopores are regularly spaced and have a narrow size distribution^51^,^52^. Here, the ordering of mesopores originates reflections at very low angles. If the wall thickness, which can be estimated by comparison with the pore sizes determined by gas sorption, is equivalent to a unit cell or less, the periodicity of the zeolite will only be retained in one dimension (along the channels), and therefore the diffraction pattern would be quite different from that of the bulk zeolite. In fact, if the reflections of the zeolite are present, as seen in the experimental pattern^51^, the material could rather comprise a physical mixture of the two phases.

The shape of reflection profiles can differ widely in HOZs, and this can be exploited to access key insights such as the size of the coherently scattering crystalline domains and the presence of lattice imperfections and strain^52^,^53^. Nonetheless, few studies have attempted to extract this information by applying line profile analysis techniques. This could be related to experimental factors such as the need for high quality data and a precise knowledge of the instrumental contributions, or just to the magnitude of the challenge posed by such extreme line broadening. In this regard, the atomic pair distribution function, which is based on the analysis of interatomic distances via a total scattering approach, promises to become an invaluable tool once fully developed. It takes Bragg and diffuse scattering into account and so is applicable to both amorphous and crystalline materials. Its value has already been demonstrated in the analysis of the delaminated zeolite ITQ-2 (ref. 54).

However, it is essential to seek complementary information to assess the full structure of HOZs. For example, the absence of a unique stacking direction combined with the extremely small crystalline regions in single crystalline mesostructured zeolite nanosheets, such as those that can be attained by, for example, repetitive branching or templating strategies, can make it impossible to detect the intergrowths by XRD^42^,^44^,^45^,^46^,^47^. Here, high resolution transmission electron microscopy (TEM) can best expose the stacking faults, twinning and the nature of the mesopores between the crystals (Fig. 4c), playing an essential role in understanding the growth mechanism. Furthermore, because electrons interact more strongly with solids than X-rays, electron diffraction extends the analysis of crystalline materials to much smaller domain sizes, which has been exploited to solve the structures of complex intergrown zeolites as ITQ-39 (Fig. 4d)^55^. The development of the automated diffraction tomography and rotation electron diffraction methods have greatly increased the accessibility and efficiency of electron crystallography (Fig. 4e), and has been applied to confirm the 3D crystalline structure of HOZs^41^,^56^.

## Nature and location of active sites

**Figure 5:**
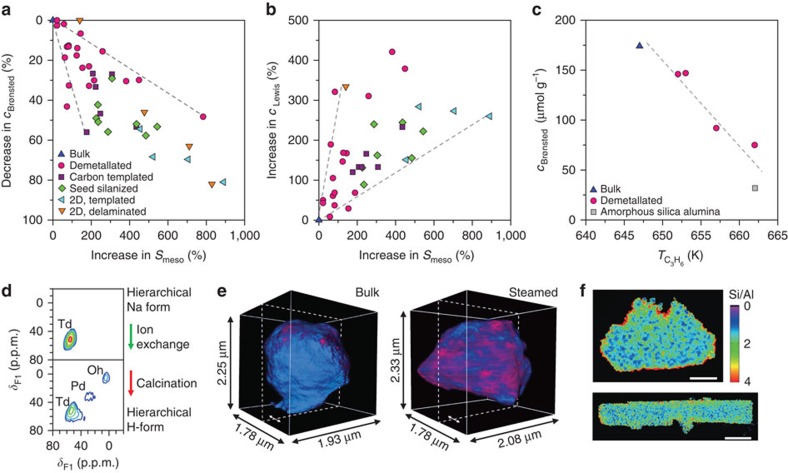
Active site distribution in HOZs. (**a**,**b**) A recent literature survey revealed a prominent reduction in the concentration of Brønsted acid sites (**a**) and an increase in the concentration of Lewis acid sites (**b**) quantified by the IR spectroscopy of adsorbed pyridine with the increasing mesopore or external surface area (*S*_meso_) in HOZs independent of the framework type or synthesis route of the zeolite, suggesting a common explanation. (**c**) Concomitant reductions in the strength of Brønsted acid sites have also been evidenced by the higher temperature required to decompose adsorbed *n*-propylamine into propene and ammonia with decreasing *c*_Brønsted_ in demetallated ZSM-5 zeolites. (**d**) ^27^Al multiple quantum NMR spectroscopy clearly distinguishes the underlying changes in the aluminium speciation, revealing that changes in the coordination are only observed upon calcination to the protonic form and not upon mesoporosity introduction by demetallation. (**e**) The changes in aluminium coordination within a bulk ZSM-5 zeolite upon steaming were spatially resolved with 30 nm resolution within 3D tomographic reconstructions attained by scanning transmission X-ray tomography with fourfold (bulk) or four-/fivefold (steamed) and sixfold coordinate aluminium (coloured blue and red, respectively). (**f**) Scanning transmission electron microscopy energy-dispersive X-ray spectroscopy maps revealed a 2.5-fold variation in the Si/Al ratio across a zeolite X-nanosheet prepared by repetitive branching (scale bars, 100 nm, top of panel; 200 nm, bottom of panel). (**a**–**f**) Adapted from ref. 53 (**a**,**b**,**d**, © 2015 John Wiley and Sons Inc), 18 (**c**, © 2013 Elsevier), 62 (**e**, © 2013 John Wiley and Sons Inc.) and 45 (**f**, © 2014 John Wiley and Sons Inc.).

The optimization of the active site quality is crucial to maximize the catalytic benefit achievable over HOZ catalysts and demands a thorough understanding. In bulk zeolites, the design of active sites primarily concerns controlling the amount and siting of negatively charged AlO_4_^−^ tetrahedra within the framework and the identity of the charge-compensating cations, which together determine the concentration and strength of Brønsted and Lewis acid sites. Comparatively, even if the crystal structure is fully preserved, it is easy to imagine that subtle distinctions may arise in HOZs, first because active sites associated with the large additional surface of the auxiliary pore network may have substantially different geometric and electronic environments from those located within the micropores, and second because changes in the aluminium speciation may be overlooked due to a limited sensitivity of analytical techniques. Significantly, a recent literature survey revealed a prominent reduction in the concentration of Brønsted acid sites in HOZs, with increasing mesoporous or external surface area independent of the framework type or synthesis (Fig. 5a,b)^53^. Furthermore, a linear relation was also observed between the strength and the concentration of Brønsted acid sites in HOZs prepared by demetallation (Fig. 5c)^18^. While early studies typically attributed distinctions in the acidic properties to the specific synthesis strategy, strong evidence attained by monitoring the evolution during successive preparation steps, indicates that the changes are most likely related to a reduced hydrothermal stability of framework aluminium in HOZs^53^,^57^.

Similarly to their bulk counterparts, the type, concentration and strength of the acid sites in HOZs are usually assessed through the infrared (IR) study of adsorbed pyridine. In the case of HOZs, however, the distribution of active sites between the internal and external zeolite surfaces requires special attention in view of the potentially more prominent catalytic role of the latter, particularly in transport or access limited reactions. This is typically approached by exploiting substituted bases of different size to selectively probe, for example, by IR or NMR, acid sites of differing size^18^,^58^,^59^,^60^, a powerful concept first developed in the form of the accessibility index^60^. However, this type of comparative analysis poses some challenges, as the range of acid sites sampled may also vary due to the distinct basicity of the probe molecules^18^. This is particularly relevant since an ideal base would detect only those sites having the required strength to catalyse the application of interest. The impact of the probe basicity may not be noticed in access limited reactions with relatively weak acidity demands, such as decalin cracking and the benzylation of toluene over MFI-type zeolites^18^,^59^, where direct correlations have been demonstrated between the concentration of acid sites at the external surface and the conversion achieved over HOZs. In contrast, distinct trends have been observed for transformations requiring stronger acid sites, such as the isopropylation of toluene or the esterification of benzyl alcohol with hexanoic acid over MFI-type zeolites^18^, where the probe molecule applied to quantify the amount of external acid sites (2,6-di-tert-butylpyridine) could not discriminate the relevant changes in the acid strength. Thus, the characterization of the acidity of the external surface remains an important challenge to enable optimization of the design of HOZ catalysts for diffusion-constrained processes.

The direct observation of the coordination of aluminium in HOZs by solid state NMR spectroscopy has been pursued to understand the structural origin of the acidity changes, commonly revealing the presence of penta- or octahedrally coordinated (non-framework) sites when the concentration of acid sites is reduced (Fig. 5d)^53^,^61^. However, the extensive broadening often observed in dehydrated samples due to quadrupolar interactions often hinders the precise quantitative comparison. Another major limitation of these bulk techniques is the lack of spatial resolution, from which it is impossible to gain insights into the relative distribution of active sites within the zeolite crystals. In pioneering work, Aramburo *et al.*^62^ recently demonstrated the application of scanning transmission X-ray microscopy to chemically probe the interior of micro-sized zeolite particles. In this way, they were able to follow the changes in quantity and coordination of aluminium upon steaming with a spatial resolution of ∼30 nm (Fig. 5e). Extending to higher resolutions, Khaleel *et al.*^45^ studied the Si/Al gradients within faujasite nanosheets by scanning TEM energy dispersive X-ray spectroscopy, which evidenced a 2.5-fold variation across the nanosheet (Fig. 5f). Understanding the catalytic impact of heterogeneities in the distribution and speciation of aluminium evidenced will undoubtedly be the subject of major future breakthroughs.

## Pore architecture

Ultimately, the benefits of hierarchical organization in zeolites depend on our ability to precisely engineer the pore architecture and correspondingly the mass transfer properties for a targeted catalytic application. A hierarchical pore structure can be defined by three primary pillars, that is, the amount, the location and the connectivity of pores associated with each level. By correctly balancing these properties, an auxiliary pore systems should illuminate the microporous domains enabling their optimal function. Porosity analysis in HOZs is a multidimensional task encompassing the micropores and any meso-/macropores associated with the zeolite crystal in addition to the intercrystalline porosity of the zeolite phase as well as that further defined upon shaping into technical form. This section examines key aspects, assessing both the applicability and strategies to extend traditional methods as well as advanced multitechnique approaches, which can be exploited to gain insight into each of these critical parameters.

### Size and amount

Early attempts to optimize the application-oriented design of HOZ catalysts were grounded on rationalizing the performance by the most directly quantifiable means, that is, the increase in the mesopore surface area of volume. As mentioned, due to the ubiquity of diffusion constraints, large activity enhancements could be readily demonstrated over HOZs in transformations involving bulky substrates and/or liquid-phase applications. For shape selective reactions, or those requiring well-defined acidic properties, it was quickly realized that extreme caution was required to ensure that the synthesis protocols preserved the integrity of the microporous domains. As such, one of the most successful descriptors for the performance of HOZs to date, the hierarchy factor and variants thereof, balanced these effects by factoring the relative mesopore surface area and micropore volume^63^. The ability to generically categorize zeolites of any framework type by readily assessable porosity characteristics was invaluable.

**Figure 6:**
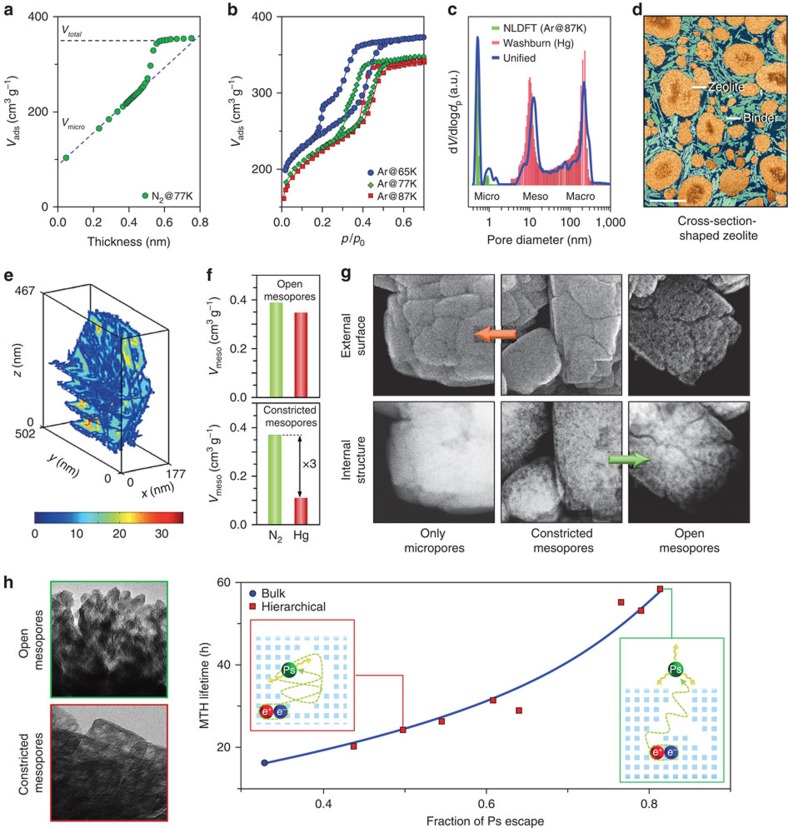
Toolbox for porosity assessment in HOZs. (**a**) The inadequacies of the *t*-plot method were recently exposed by the underestimation of the micropore volume in a mesostructured USY zeolite. (**b**) Argon isotherms evidence the hysteresis induced in small mesopores within a mesostructured USY zeolite upon measurement at lower temperatures. (**c**,**d**) A unified model connecting the pore size distributions derived from argon sorption and mercury porosimetry in a shaped zeolite catalyst (**c**) emphasizes the importance of integrative data analysis approaches. A cross-sectional focused ion beam scanning electron tomography image clearly reveals the internal particle organization originating the multimodal porosity (scale bar, 2 μm (**d**)). (**e**) The size of the micropore domains in HOZS are quantified by TEM tomography. (**f**,**g**) The accessibility of open and constricted mesopores in hierarchical ZSM-5 zeolites can be distinguished from the comparative mesopore volume determined by Hg intrusion and N_2_ sorption (**f**). Identical-location scanning electron and scanning transmission electron micrographs reveal the structural origin (scale bar, 100 nm (**g**)). (**h**) The direct correlation and the methanol-to-hydrocarbon (MTH) lifetime of hierarchical ZSM-5 zeolites demonstrates the unique sensitivity of positron annihilation lifetime spectroscopy to the pore connectivity and related function of the auxiliary pore network. As illustrated schematically, the presence of open or constricted mesopores observed by TEM (scale bar, 20 nm) strongly impacts the amount of ortho-positronium escaping from the zeolite. (**a**-**h**) Adapted from ref. 64 (**a**, © 2014 American Chemical Society), 56 (**b**, © 2014 John Wiley and Sons Inc.), 74 (**c**,**d**, © 2015 American Chemical Society), 66 (**e**, © 2012 John Wiley and Sons Inc.), 22 (**f**,**g**, © 2014 Macmillan Publishers Ltd) and 28 (**h**, © 2015 John Wiley and Sons Inc.).

While both N_2_ and Ar sorption are popular methods for the textural characterization of HOZs (Fig. 6a–c), monatomic Ar is often preferred due to its smaller kinetic diameter (0.34 versus 0.37 nm), higher adsorption temperature (87 versus 77 K) and weaker fluid–wall interactions than diatomic N_2_, which help to reduce potential diffusion limitations and shift the adsorption within the micropores to higher pressures, respectively. In this respect, there has been little innovation on the traditional models applied to bulk zeolites. However, standard methods to access key structural parameters from N_2_ isotherms, such as the Brunauer–Emmett–Teller), *t*-plot and BJH (Barrett–Joyner–Halenda) methods, were developed using reference isotherms from non-porous materials. The inadequacy of these assumptions was recently demonstrated by the revelation that the *t*-plot analysis could lead to up to a 40% underestimation in the micropore volume^64^ (Fig. 6a). The difficulties associated with the derivation of pore size distributions (BJH) from N_2_ desorption, which often results in phantom contributions at 4 nm thought to depend on the pore connectivity, have also been extensively described^65^. Comparative analysis of the adsorption and desorption branches readily demonstrates this problem^66^ and enables estimation of the volume of occluded or constricted mesopores. More advanced implementations of the *t*-plot and BJH method generic to both nitrogen or argon isotherms have been proposed^67^,^68^,^69^, which remove the ambiguity of using the traditional BJH method with argon isotherms^38^.

For both bulk and HOZs, modern pore modelling techniques based on non-localized density functional theory (NLDFT) are now routinely adopted for the assessment of micro- and mesopore sizes, volumes and surface areas in a single approach, yielding a good agreement with Brunauer–Emmett–Teller surface area and capillary condensation^70^. Nevertheless, these approaches may be in danger of becoming too ‘press button'. Potential differences in the sorption properties of HOZs have yet to be rigorously addressed. Preliminary studies by Cho *et al.*^52^ revealed distinctions in the monoclinic–orthorhombic phase transition in an *in situ* XRD study of the structural changes during Ar sorption. Similarly, distinctions in the hydrophobicity evidenced by water sorption highlighted that the surface of the secondary pore networks should not be approached as identical to those of bulk zeolites^71^. Non-standard probes as hydrocarbons or water would no doubt find extended use for porosity characterization with the development of improved kernels for data analysis, and chemical interactions of the probe with the zeolite could be turned to advantage to gain further insight into the surface properties.

Mercury porosimetry offers a highly complementary tool^22^,^72^,^73^ for the comprehensive description of auxiliary pore systems integrating pores >4 nm in diameter, and becomes of paramount importance during the scale-up of zeolite catalysts into macroscopic-shaped forms. In principle, by coupling the information derived from Hg porosimetry and gas sorption, it is possible to access the pore size distribution across the entire range of length scales (Fig. 6c), the organization of which can be clearly visualized by FIB-SEM. Yet remarkably, until recently, no unified model had been developed to bridge the data from sorption isotherms and intrusion curves^74^. The convergence marks a transition from technique specific to unified descriptions of the pore structure, illustrating a clear direction for the development of new methods to gain insight into materials with hierarchical pore structures.

### Location

Given the structural diversity of HOZs, a knowledge of the bulk porous properties alone is insufficient to establish crucial aspects regarding the integration of auxiliary pore systems, for example, whether the different porosity levels coexist in the same phase, how the pores are distributed and so on. In this regard, nothing is more tangible than the direct visualization of the pore architecture^75^. Compared with bulk zeolites, where microscopic techniques are primarily used to determine the particle size and morphology, the multidimensional challenge of examining the structural organization in HOZs has fuelled the development of much more advanced imaging approaches^66^,^72^,^76^,^77^. Fronting this movement, de Jong *et al.* elegantly exemplified the 3D TEM tomographic reconstruction and quantitative analysis of the pore network within commercial USY zeolite crystals^66^,^76^. While this enabled the derivation of the fraction of constricted mesopores, the tortuosity of the mesopore network and the size distribution of the remaining microporous domains (Fig. 6e)^66^, the catalytic relevance of these parameters has yet to be widely explored. At a similar time, Karwacki *et al.*^77^ took a different strategy, demonstrating the application of focused ion beam scanning electron microscopy to examine, with ∼5.2 nm resolution, the heterogeneity of the size of mesopores developed upon steaming with respect to the crystallographic orientation in individual coffin-shaped ZSM-5 crystals (100 × 20 × 20 μm^3^). Although potentially yielding valuable mechanistic clues to mesopore formation, it is unclear how these insights would translate to the smaller particle sizes and/or more complex morphologies typical of industrial zeolites, which would be difficult to directly examine by this method.

It is clear that optimal imaging approaches must deliver the right information while permitting a high throughput to enable good statistical representativeness. Thus, until improved methods are developed to automate image acquisition, tilt series alignment, reconstruction, and visualization and interpretation^78^, tomographic methods will likely not gain wide applicability for routine analysis. A bridge might come in the form of correlative strategies. For example, in model MFI-type zeolites of equivalent bulk porosity, the integration of the mesopores in distinct ‘open' and ‘constricted' configurations could be corroborated on the basis of a multitechnique assessment. Herein, the difference in mesopore volume determined by the application of mercury porosimetry in conjunction with nitrogen sorption, provided a measure of the accessibility of the auxiliary pore network (Fig. 6f). Further insight into the structural origin of the differing accessibility was attained through the simultaneous observation of the external and bulk structure by identical location secondary electron and high angle annular dark field imaging (Fig. 6g)^22^, which confirmed the more internal or external location of the mesopores. Of course electron-based techniques have well known limitations, such as the need for beam transparency in transmission modes, and the structural sensitivity of the sample to electron irradiation as well as to the cutting methods applied during the sample preparation and/or image acquisition. Here, strategies to minimize the sample exposure, such as through the use of scanning or low dose imaging modes, and to increase the sample tolerance, for example, through cryogenic cooling, are becoming increasingly popular^79^.

### Connectivity

Independently of the amount or location, if the auxiliary pore networks are poorly connected they will not efficiently contribute to an improved mass transfer in HOZs. Connectivity is a complex multivariate parameter that embraces the interfaces between each porosity level, that is, the micro-mesopore, micro-macropore and meso-macropore unions. While the visualization and porosimetry approaches described capture certain features of the connectivity of the secondary pore systems, the most interesting aspect of the micro- and mesopore interface remains tantalizingly out of reach.

Although manifested in the response of numerous methods, so far very little progress has been made to quantitatively discern the impacts of pore connectivity. A clear example is that of gas sorption. Moving away from the standard analysis, recent work by Garcia-Martinez *et al.*^56^ demonstrated the potential to probe pore constrictions in a mesostructured USY zeolite via scanning the hysteresis loop in the Ar isotherms. While reversible pore adsorption was evidenced at 87 K, by lowering the temperature (77 and 64 K) it was possible to induce and thereby study the form of the hysteresis loops (Fig. 6b). Similar indications of the sensitivity of gas sorption were also attained by molecular simulations of Coasne *et al.*^80^, which suggest that the configuration of the hierarchical pore structure could disrupt capillary condensation, suppressing the hysteresis loop in N_2_ isotherms. However, it is important to note that hysteresis loops may also be suppressed by surface roughness as well as temperature, and the lack of hysteresis may be misinterpreted as a well-connected pore structure.

Only very recently, positron annihilation lifetime spectroscopy was shown to have unprecedented sensitivity to the pore architecture in HOZs^22^,^28^. Remarkably, a direct link between the escape of ortho-positronium species formed upon positron implantation to vacuum, which directly relates to the global connectivity of the pore network, and the catalyst lifetime of hierarchical MFI-type zeolites was established in the conversion of methanol to hydrocarbons (Fig. 6h)^28^. The dynamic way in which positrons interrogate a porous solid opens unique opportunities for the characterization of their pore architecture and chemical functionality^81^,^82^,^83^,^84^. However, this work is currently in very early stages and a number of practical factors, such as the need for a radioactive source and the lack of a comprehensive framework for spectral analysis and interpretation, need to be addressed to widen the applicability of the technique for the assessment of HOZs.

Notably, the sensitivity of positron annihilation lifetime spectroscopy was only initially recognized through the comparison of HOZs purposefully synthesized with distinct mesopore locations. This suggests that, with an improved understanding, there is still room for other less well known techniques to contribute to the growing demand for a precise structural understanding of hierarchical materials. For example, hyperpolarized ^129^Xe NMR^85^,^86^, which was previously shown to be sensitive to the exchange between the different pore environments in both delaminated and mesoporous zeolites, could yet offer a more tangible means to rationalize mass transfer behaviour. Since pore connectivity is among the main parameters influencing molecular transport, diffusion measurements of the actual substrates and products, can, in turn, be expected to contribute to the elucidation of pore connectivity. For example, the enhanced oil-to-gasoline conversion and reduced tendency to coke formation in various fluid catalytic cracking catalysts with increasing diffusivities, closely corroborates the relation between the catalyst lifetime and the pore connectivity^87^.

## Guest diffusion and catalyst effectiveness

**Figure 7:**
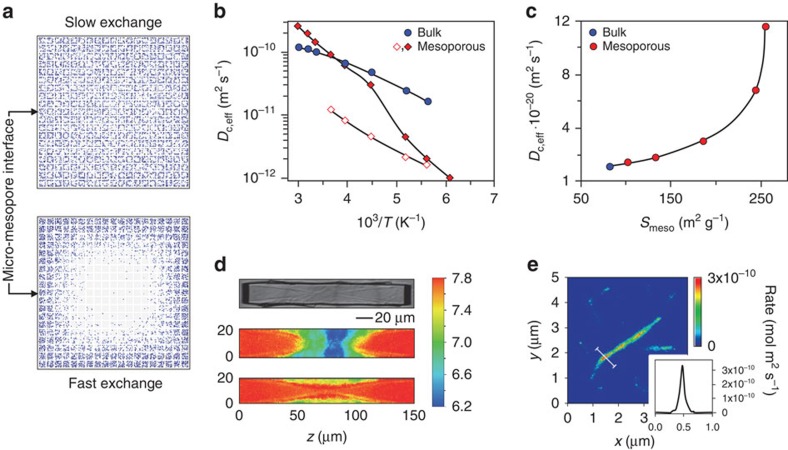
Enhanced diffusion properties and catalyst effectiveness. (**a**) Snapshots determined by dynamic Monte Carlo simulations illustrate the different guest distributions expected within zeolite crystals under the limiting cases of slow and fast exchange between the meso- and micropores. (**b**) Self-diffusivity of ethane in bulk and mesoporous LTA zeolites determined by pulsed field gradient NMR. Mass transfer strictly adheres to normal diffusion over the space and timescales of the measurements. Even at high temperature, ethane diffusion in the mesoporous zeolite does not significantly exceed that in the bulk counterpart, while at low temperature the similarly reduced values reflect the low amount of molecules in the mesopores. Blockage of the mesopores with cyclohexane (open symbols) is evident from the reduced, but equivalent temperature dependency of the ethane diffusivity compared with the bulk zeolite. (**c**) Correlation of the effective diffusivity, determined by gravimetry under slow-exchange conditions, of 2,2-dimethylbutane in hierarchical ZSM-5 with the interface area *S*_meso_. The sharp increase evidenced above 150 m^2^ g^−1^ was ascribed to the attainment of an interconnected mesopore network. (**d**) Concentration profiles of benzene within MFI-type zeolite crystals are monitored along different crystallographic directions by measuring the change in the refractive index. (**e**) The catalytic conversion of furfuryl alcohol is mapped within single-needle-shaped ZSM-22 zeolite crystals with nanometre accuracy by fluorescence microscopy. The inset shows the reaction rate as measured along the white line. (**b**–**e**) Adapted with permission from ref. 105 (**b**, © 2012 Elsevier), 89 (**c**, © 2014 John Wiley and Sons Inc.), 95 (**d**, © 2014 Macmillan Publishers Ltd) and 103 (**e**, © 2009 © 2015 John Wiley and Sons Inc.).

The primary aim of designing HOZs is to utilize active sites more efficiently in catalytic applications by improving the transport attributes, which can be expressed in terms of the catalyst effectiveness. Effectiveness factors and the expected concentration profiles of guests within zeolites can be estimated with knowledge of the effective diffusivity and some basic kinetic parameters. Depending on the rate of exchange between the micropores and the auxiliary pore network, two limiting cases can be identified that determines the relative contribution of mesopore diffusion (fast exchange) and the reduction in the characteristic diffusion length (slow exchange) to the overall transport enhancement of a given HOZ (Fig. 7a). Yet, although the superior performance of HOZs is commonly ascribed to facilitated molecular transport, until recently only a limited number of studies had measured the diffusion characteristics. Transport enhancements can in principle be simply accessed through any method capable of following the rates of uptake and release of a guest molecule, such as gravimetry^46^,^88^,^89^,^90^, IR or NMR spectroscopy^90^,^91^, or the zero-length column and frequency response methods^90^,^91^. However, in practice, variations in the absolute values and relative enhancements of the effective diffusivity over orders of magnitude are not uncommon depending on the technique, measurement conditions and probe molecules used, as well as the sample properties (Fig. 7b,c). In fact, for a given zeolite and hierarchical pore structure, the prevailing mechanism of intrinsic mass transfer can be altered by the guest molecules and the measurement conditions.

With respect to the sample, two particularly challenging aspects are dealing with the heterogeneity of the crystal and of the mesopore size and geometry, the effects of which may completely dominate over the governing mechanisms of mass transfer. Notably, due to the prevalence of transport resistances at the external surface^92^ or within the zeolite crystal^93^,^94^, even the mass transfer in bulk zeolites cannot be described by an intracrystalline diffusivity alone^90^,^91^. The situation only becomes more complex for HOZs, in which interfacial barriers between the additional porosity levels must also be accounted for. If the extension of the micropore domain is much smaller than that of the crystal, the contribution of such barriers to the overall transport could dominate over the resistance by micropore diffusion. Consequently, to attain any information about the microdynamic origins of transport enhancements, a highly involved analysis requiring dedicated expertise is normally essential.

To achieve the dream of predicting the concentration profiles of molecules within the pore networks, the in-depth exploration of the elementary processes of mass transfer through the application of microscopic diffusion measurements is essential. IR microimaging (Fig. 7d)^95^ as well as single molecule tracking^96^,^97^ and microspectroscopy^98^, can potentially distinguish the two limiting cases of guest distribution in HOZs. While single molecule tracking provides direct information about the rate of propagation of individual molecules, and thus the permeability of the pore spaces^96^, quasielastic neutron scattering^90^,^99^ and pulsed field gradient NMR^90^,^91^ offer complementary insights concerning whole molecular assemblies. Being applicable to any proton containing molecule, the range of probes amenable to the latter techniques significantly exceeds that of single molecule tracking, which so far necessitates the application of fluorophores. While the diffusion path lengths accessible by pulsed field gradient NMR vary from approximately a hundred nanometres to tens of micrometres, quasielastic neutron scattering is sensitive to displacements over nanometres enabling the rate of molecular displacements within the purely microporous regions to be monitored even in hierarchical pore systems. Complementing information about molecular exchange rates between different pore spaces may be provided by ^129^Xe NMR^85^,^86^ and solid state exchange NMR^100^ by exploiting their sensitivity to the chemical environment. Simulation studies searching of the structural origin of these resistances are also gaining increasing relevance^101^,^102^.

Though we now dispose of highly sophisticated techniques to quantify transport enhancements by measuring the uptake and release of guest molecule, the correlation with the catalytic properties of HOZs is still rather limited. These difficulties are largely related to the nanoscopic size of the crystalline domains in HOZs, given the fact that the monitoring of transient concentration profiles during mass separation and catalytic conversion has only quite recently become possible by microimaging in large bulk crystals^95^. Knowledge of such profiles, however, is a prerequisite of any in depth understanding of mass transfer phenomena, and it is expected that advancements will enable extension of the techniques to match or go beyond the reactivity mapping achieved through fluorescence microscopy (Fig. 7e)^97^,^103^.

## Future directions

This review has demonstrated that the structural analysis of HOZs should not simply be approached in a similar manner to their bulk predecessors. New strategies are essential to access vital information about their crystal, active site and pore structure that is necessary to rationalize their performance. Several actions are viewed as critical to move forward with the design. Beginning with the synthesis, the attainment of an improved understanding of key mechanistic aspects, including the implications of integrating auxiliary pores at the atomic scale and the pore network organization at the mesoscale, will undoubtedly guide the development of more efficient zeolite catalysts. Theoretical studies are expected to hold great potential here, as approaches are devised to overcome the challenges posed by the system complexity.

As highlighted, the scope of established techniques requires careful reassessment to ensure the adequacy for the characterization of materials with multilevel pore structures, and, where necessary, new and improved methods to assess primary structural attributes need to be developed. For example, due to the dominant catalytic role in diffusion constrained reactions, a greater emphasis on the characterization of the active sites associated with the surface of the auxiliary pore network will be invaluable. Similarly, advanced methods are required to comprehensively analyse the impact of reductions in the coherent domain size on the crystal structure. Coupled to this, the wider comparative catalytic assessment of zeolites with different morphologies, which has so far only be tackled by a limited number of studies^22^,^44^,^104^, is essential to confirm the relative effectiveness. Herein, the reproducibility of the synthesis procedures between labs will be essential to corroborate the findings. Integral to the understanding and quantification of the improved catalyst effectiveness in HOZs, the advancement of methods to monitor guest profiles constitutes a primary challenge of future research.

Given the complexity of these multidimensional systems, intensive interdisciplinary research involving the development of integrated approaches and the comparison of model samples will be crucial to accomplish many of these tasks. Similarly, improved methods of data analysis and the wider accessibility of advanced analytical techniques will greatly facilitate the identification of structure–performance relations. We expect that many of the important aspects raised through the examination of HOZ catalysts will find broad general relevance in the design of other hierarchically organized materials.

## Additional information

**How to cite this article:** Mitchell, S. *et al.* Structural analysis of hierarchically organized zeolites. *Nat. Commun.* 6:8633 doi: 10.1038/ncomms9633 (2015).

## Figures and Tables

**Figure 1:**
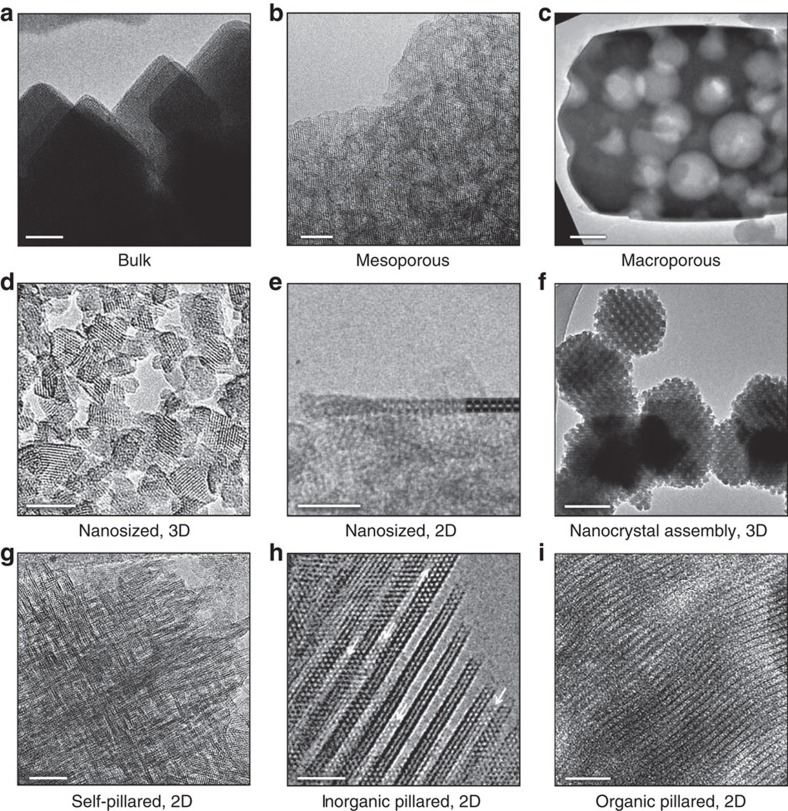
Hierarchical organizations in zeolites. (**a**–**i**) Compared with a bulk material (**a**), TEM micrographs illustrate the distinct ways in which zeolites can be furnished with hierarchical pore structures. Both bottom-up and top-down synthesis approaches can be followed to configure the secondary porosity either within (intracrystalline, **b** and **c**) or between (intercrystalline, **d**–**i**) the zeolite crystals; a mesoporous USY zeolite attained by demetallation (**b**), a macroporous MFI-type zeolite prepared by steam-assisted crystallization (**c**), a nanosized Y-zeolite directly synthesized by a non-templated approach (**d**), an ITQ-2 zeolite derived by delamination of MCM-22 (**e**), an intergrown assembly of spherical silicalite-1 nanocrystals attained by confined synthesis in a mesoporous carbon template (**f**), a self-pillared assembly of ZSM-5 lamellae prepared by repetitive branching (**g**), silica-pillared ZSM-5 nanosheets synthesized by surfactant templating (**h**) and an organic–inorganic-layered hybrid with organic linkers covalently bonded to ICP-1P zeolite layers (**i**). This review examines the state of the art in the structural analysis of these morphologically diverse materials with the aim of establishing directions for their improved design in catalytic applications. Scale bars, 20 nm (**a**,**b**,**g**,**i**), 200 nm (**c**,**f**), 10 nm (**d**,**e**,**h**). (**a**–**i**) Reprinted with permission from ref. 22 (**a**, © 2014 Macmillan Publishers Ltd), 105 (**b**, © 2011 American Chemical Society), 106 (**c**, © 2015 John Wiley and Sons Inc.), 49 (**d**, © 2015 Macmillan Publishers Ltd), 107 (**e**, © 1998 Macmillan Publishers Ltd), 108 (**f**, © 2011 American Chemical Society), 44 (**g**, © 2010 AAAS), 37 (**h**, © 2010 American Chemical Society) and 109 (**i**, © 2014 American Chemical Society).

**Figure 2:**
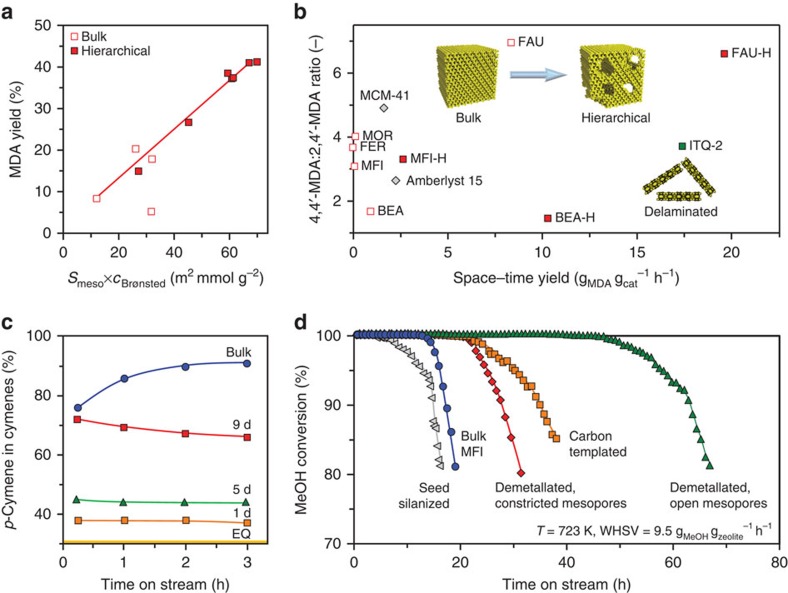
Opportunities and threats for zeolite catalysis. (**a**) The linear correlation between the yield of methylenedianiline (MDA) mixtures and the mesopore surface area factored by the concentration of Brønsted acid sites evidenced over FAU-type zeolites in the liquid phase condensation of aniline with formaldehyde to MDA, a key intermediate in polyurethane production, exemplifies the need to balance active site and pore quality to maximize their performance. (**b**) Compared with other zeolite framework types, the activity of the FAU-type catalysts could be significantly enhanced while retaining the unique shape selective properties by preserving the crystal structure and alleviating the diffusion constraints. (**c**) Evaluation of nanosized (2D) MFI-type zeolites hydrothermally synthesized for different durations (1–9 d) in the alkylation of toluene with isopropanol reveals that at least two pentasil layers (9 d) are required to substantially increased para-selectivity with respect to that expected thermodynamically (equilibrium, EQ), although this remained inferior to that observed over the bulk zeolite. (**d**) The key role of both pore and active site quality was also demonstrated by the varying catalyst lifetimes evidenced in the conversion of methanol to hydrocarbons over hierarchically organized MFI-type catalysts of equivalent bulk composition, but synthesized by different approaches. (**a**–**d**) Adapted with permission from ref. 16 (**a**,**b**, © 2015 American Chemical Society), 19 (**c**, © 2013 The Royal Society of Chemistry) and 22 (**d**, © 2014 Macmillan Publishers Ltd).

**Table 1:** Characterization of the key attributes of HOZ catalysts.

**Property**	**Technique**	**References**
Crystal structure	Powder X-ray diffraction with line profile analysis or a related approach	29,30,52,53,54
	High resolution transmission electron microscopy	42,44,45,46,47,48,49
	Electron diffraction and electron diffraction tomography	41,56
Acid site structure	Temperature-programmed desorption of adsorbed probesIR spectroscopy of adsorbed probes such as substituted pyridines and CO	18,18,26,57,58,60
	NMR spectroscopy including adsorbed probes such as organophosphine oxides	53,59,61
	Energy dispersive X-ray spectroscopy	45,72,88
	Scanning transmission X-ray microscopy	62
Pore structure	Ar or N_2_ sorption including scanning measurements at different temperatures	52,65,74
	Hg porosimetry	22,72,73,74
	Adsorption of other probes as water or hydrocarbons	71
	Synchrotron X-ray tomography	72
	Focused ion beam scanning electron tomography	72,77
	Scanning and transmission electron microscopy including identical location	22,106,107,108,109,110
	3D electron tomography	66,75,76
	Positron annihilation lifetime spectroscopy	22,28
Mass transfer properties	Solid state NMR including pulsed field gradient	85,90,91,100
	Quasi elastic neutron scattering	90,99
	Gravimetric methods	88,89
	Chromatographic methods including the zero-length column	90,105
	IR microimaging, single molecule tracking and microspectroscopy	95,96,97,98,111

3D, three dimensional; HOZ, hierarchically organized zeolite; IR, infrared; NMR, nuclear magnetic resonance.
